# Chandipura Virus infection in mice: the role of toll like receptor 4 in pathogenesis

**DOI:** 10.1186/1471-2334-12-125

**Published:** 2012-05-29

**Authors:** Balakrishnan Anukumar, Prajakta Shahir

**Affiliations:** 1 National institute of Virology Kerala Unit, 2nd Floor, E-Block, T.D. Medical college hospital complex, Vandanam, Alappuzha, Kerala, 688005, India

## Abstract

**Background:**

The susceptibility of mice and humans to Chandipura virus infection is age-dependent. Upon experimental infection, mice secrete significant amounts of proinflammatory cytokines. Similarly, children who recover from natural infection with the virus show significant amounts of TNF-α production, suggesting that innate immunity plays a major role in the response to Chandipura virus. Toll-like receptors (TLR) are key host molecules involved in innate immune responses in infections. Therefore, the aim of this study was to examine the role of TLR in the response to Chandipura virus infection.

**Methods:**

The mouse monocyte-macrophage cell line, RAW 264.7, and C3H/HeJ mice were used as models. Micro array techniques were used to identify the type of TLR involved in the response to infection. The results were validated by examining TLR expression using flow cytometry and by measuring the levels of proinflammatory cytokines and nitric oxide (NO) in the culture supernatants using bead assays and the Griess method, respectively. The pathogenic role of Toll-like receptor 4 (TLR4) was studied in a TLR4 mutant strain of mice -C3H/HeJ and the results compared with those from wild-type mice- C3H/CaJ. The pathogenic effects of NO were studied by treating experimentally infected mice with the NO inhibitor, aminoguanidine (AG).

**Results:**

The micro array results showed that TLR4 was regulated after Chandipura virus infection. At high multiplicities of infection (10 MOI), RAW cells up- regulated cell surface expression of TLR4 and secreted significant amounts of TNF-α, MCP-1, IL-10 and IL-12 and NO. The survival rate of C3H/HeJ mice was higher than those of wild-type C3H/CaJ mice. The survived C3H/HeJ mice secreted significant quantity of MCP-1 and IFN-γ cytokines and cleared virus from brain. Similarly, the survival rate of AG-treated mice was higher than those of the untreated controls.

**Conclusions:**

Chandipura virus regulates TLR4, which leads to the secretion of proinflammatory cytokines and NO by infected RAW cells. Difference in survival rate in TLR4 mutant mice and nitric oxide inhibitor treated mice, confirmed the role of these molecules in disease pathogenesis. The result is significant in clinical management and designing antiviral intervention for Chandipura virus infection.

## Background

Chandipura virus (CHPV) belonging to the family *Rhabdovirdae*, genus *vesiculovirus* has been associated with acute and fatal encephalitis of young children in several parts of India [1,2]. Children under 15 years of age are vulnerable to natural infection, whereas adults are refractory. Similarly, susceptibility studies in mice show that CHPV is lethal to young mice, but adults are only susceptible through the intra-cerebral route of infection [3]. This age-dependent susceptibility is also apparent in nude mice [4]. Further, virus-infected young mice secrete significant amounts of proinflammatory cytokines [5] and children who have recovered from natural infections secrete significant levels of TNF-α [6]**.** Taken, together, these observations strongly suggest that innate immunity plays a pivotal role in protection from CHPV infection. The role of innate immunity is critical during early childhood, particularly when humoral and cell mediated immune responses are not fully developed.

Innate immunity is mediated by several mechanisms. Toll-like receptors (TLR) are key host molecules involved in innate immune responses to infection [7]. TLRs were first identified in Drosophila. To date, 13 mammalian TLRs have been identified [8]. Toll pathways are known to respond to Gram positive bacterial and fungal infections, and are essential for anti-viral immunity [9]**.** In humans, TLR1, TLR2, TLR4, TLR5 and TLR6 are cell membrane-associated, and respond primarily to bacterial surface-related pathogen associated molecular patterns (PAMPs). A second group, comprising TLR3, TLR7, TLR8 and TLR9, is found on the surfaces of endosomes, and responds primarily to nucleic acid-based PAMPs derived from viruses and bacteria. The interaction between TLRs and viral ligands leads to the secretion of both proinflammatory cytokines, type I interferons and nitric oxide (NO) [10,11]. Recent studies also show that several types of neural tissue cell up-regulate particular TLRs in response to infection [12]. Failure to prevent or control TLR response could lead to exuberant induction of detrimental cytokines. Various experimental mice model suggests that TLR activation promotes protective antiviral immunity in some cases, while exacerbating disease in others [13]. A role of different TLRs in the response to viruses has been established [14-19].

A growing body of evidence indicates that one particular product of the innate immune response, NO, or its derivatives, has inhibitory effects against a variety of viral infections. NO is one of the products of TLR4 stimulation [20]. Nitric oxide synthase (iNOs) mutant mice are more vulnerable to some types of infection than wild-type mice [21]. NO hinders the productive infection of several animal viruses, including herpes simplex virus 1 (HSV1) [22]**,** ectromelia virus, vaccinia virus (VV) [23]**,** vesicular stomatitis virus (VSV) [24] and murine leukemic virus (MLV) [25]**.** This suggests that NO could be one of the vital factors that enable host innate immune responses to control the initial stages of viral infections within the central nervous system. NO plays a role in host defense mechanisms due to its antibacterial and virustatic properties; however, if NO synthesis is not properly regulated, damage of host cells occurs due to its inherent cytotoxicity [26].

In the present study, we focused on the types of TLR involved in immune responses to CHPV infection using RAW cells as an *in vitro* model. The role of TLRs in disease pathogenesis is also investigated in mice. The results identify a role for TLR4 in the response to CHPV infection and its role in disease pathogenesis.

## Methods

### Cells and virus

Vero E6 and RAW 264.7 cell lines were obtained from the National Centre for Cell Science, Pune, India. The cell lines were cultured and maintained in Dulbecco’s modified Eagle’s medium (DMEM), supplemented with 10% fetal calf serum (FCS, Gibco) and antibiotics. The Chandipura virus strain (034267) was originally isolated from the CHPV outbreak in Andhra Pradesh in 2003 [2]**.** The virus was propagated and titrated in vero E6 cells.

### Viral infection

Confluent monolayer of RAW cells (≈10^6^ cells) was infected with CHPV at 10^7^ 50% tissue culture infective dose per mL (TCID_50_/mL). After adsorption for 1- h at 37°C, the cells were washed with PBS and incubated with DMEM for 24 h at 37°C with 5% CO_2_. Triplicate cultures were harvested at various times from 1 to 24 h post infection (PI); centrifuged for 5 min at 2500 xg, and virus concentration was determined by endpoint dilutions assay. The titer was calculated by the method of Reed and Muench [27].

### Microarray analysis

Total RNA was isolated from control and infected RAW cells at 5 h PI using an RNeasy mini kit (Qiagen). The quality and integrity of the RNA was checked before labeling using a 2100 Bioanalyzer (Agilent). RNA was labeled by using Agilent Low RNA Input Linear Amplification kit. Hybridization was performed on mouse microarray slides with 22000 prints (G4112A, Agilent). The biological repeat was performed on the same samples. Data were collected from the hybridized slides using GeneSpring GX 7.2 software and normalized using by Per Spot and Per Chip intensity dependent (Lowess) normalization. Up-regulated genes (ratio > 2) and down-regulated genes (ratio < 0.5) were used to calculate -fold changes. Genes showing -fold changes (log_2_) > 1 (for up-regulated genes) and > −1 (for down-regulated genes) were further analyzed. The significant functional classification of differentially regulated genes was analyzed using the GeneSpring GX 7.2 software ontology browser and differentially regulated genes significantly (*P* < 0.05) contributing to the pathways was identified. All data were MIAME-compliant and the raw data has been submitted to Gene Expression Omnibus (GEO -NCBI, Acc.No.GSE 13082).

### TLR4 expression

Cell culture dishes containing confluent monolayer of RAW cells were divided into three groups. The first group was treated with different concentrations of LPS (0–1000 ng/dish). The second group was infected with different MOI of virus (10, 1, or 0.1). The third group was used as uninfected control. All dishes were incubated for 24 h at 37°C with 5% CO_2_. The cells were then washed with PBS and scraped. Single cell suspensions were produced by slow pipetting, and the cells were pelleted by centrifugation at 300 ×g for 10 min. One million cells were suspended in 100 μL of FACS staining buffer (PBS pH 7.2, 2% FCS, 0.09% sodium azide, 1% mouse serum) and stained with an anti mouse TLR4/MD2 antibody conjugated to phycoerythrin-cyanine 7 (PE-Cy7) (eBioscience). Appropriate isotype controls were included to set the base line data. After 1-h incubation at 4°C, the cells were washed thrice with staining buffer and the pellet suspended in 500 μL of 1% paraformaldehyde. The cells were acquired and analyzed using a FACSCalibur cell sorter and Cell Quest Pro software (BD Bioscience).

Half-offset histograms were prepared using Flow Jo software (Tree Star, USA).

### Quantification of mouse inflammatory cytokines

The levels of IL-12p70, TNF-α, IFN-γ, MCP-1, IL-10 and IL-6 in the cell culture supernatants were quantified using a BD™ Cytometric Bead Array (CBA) mouse inflammation kit (BD Biosciences). The minimum/maximum sensitivity of this assay is 0–5000 pg/mL. The levels of the six cytokines were measured simultaneously in 50 μL of undiluted culture supernatant and expressed as pg/ml.

### Mice experiments

The pathogenicity study was conducted in C3H/HeJ and C3H/CaJ mice. Groups of mice (13-days-old) were used in this experiment (*n* = 8, plus the mother per group). The experimental procedure was followed as per the recommendations of the Committee for the Purpose of Control and Supervision on Experiments on Animals (CPCESA), India and the protocol was approved by the Institute Animal Ethics Committee (IAEC), National Institute of Virology, Pune. All efforts were made to minimize suffering. Both C3H/HeJ and C3H/CaJ mice were infected with 100 μL (10^5^ TCID_50_/mL) of CHPV via the subcutaneous route. The mice were observed for sickness and mortality up to 10 days PI. Blood and brain was collected at 0, 24, 48, 72 and 96 h PI. Blood was collected intra orbitally before sacrifice and the sera were separated. Mice were perfused transcardially with 20 mL of PBS and the brain was collected and frozen immediately at −80°C. Before use, the frozen brain was freeze thawed and 10% homogenate prepared in PBS. The suspension was clarified at 1000 ×g for 10 min and the virus in the supernatant was titrated. Cytometric bead array was performed from 50 μL of undiluted serum.

### IgM kinetics

IgM capture ELISA was done following procedure described for human [2] with the modification that coating was done with anti-mouse IgM as a capture antibody. The level of IgM was determined from 1/100 diluted samples. The cut off value was set by average plus 3 standard deviation of optical density (OD) from uninfected negative control mice.

### Quantification of nitric oxide

The cells were infected with different MOI (0.1, 1, and 10 MOI) of virus and the supernatants harvested at 24 h PI. NO levels were measured using the Griess method [28]. Briefly, 100 μL of Griess reagent (1% sulfanilamide, 0.1% naphthylethylene diamine dihydrochloride, 2.5% H_3_PO_4_) was added to 100 μL of cell supernatant in a 96-well plate. The colorimetric reaction was allowed to proceed for 10 min at room temperature and the OD at 540 nm (with a reference wavelength of 655 nm) was measured in a micro plate reader (Model 680 XR, BioRad). The measured OD values were converted to equivalent concentrations using a standard curve established from serial dilutions of sodium nitrite (NaNO_2_) in culture medium. All the samples were assayed in triplicate.

### NO inhibitor treatment

Swiss albino mice (13-days-old) were divided into two groups (*n* = 8, plus the mother per group). Both the groups of mice were infected with 100 μL (10^5^ TCID_50_/mL) of CHPV via the subcutaneous route. One group was injected with 100 μL (100 mg/kg body weight) of aminoguanidine (AG), and the other was used as a virus control.Treatment started at 24 h PI and continued up to 120 h PI. The mortality pattern was recorded and the percentage mortality calculated.

### Statistical analysis

The Student *t* test was used to compare the means values for the treated and control groups. A *P* value < 0.05 was considered significant. The mean survival time was calculated using Kaplan Meier statistics for survival function (GraphPad prism 5 and PASW Statistics 18 software).

## Results

### One-step growth cycle of CHPV

**Figure 1:**
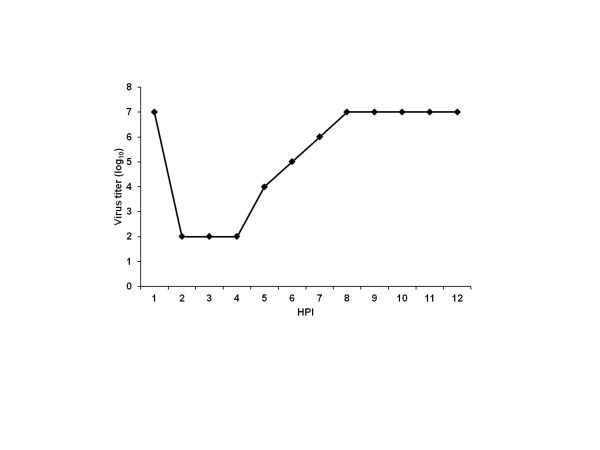
** One-step growth cycle of Chandipura virus in RAW cells.** RAW cells were infected with virus. At different time points, the culture supernatants were collected for virus titration. The supernatant was titrated in vero E6 cells and the titer was expressed as log_10_ tissue culture infective dose 50% (TCID_50_) per 100 μL. The line graph is showing virus titer up-to 12 h PI and the titer was remained same at other PI hours tested.

RAW 264.7 cells were used to identify the type of TLR involved in the immune response to CHPV infection. Initially, the susceptibility of this cell line to CHPV was confirmed using a one-step growth cycle. Infected RAW cells produced detectable levels of virions from 4 h PI, and the maximum titer was observed at 8 h PI (Figure 1).

### Regulation of TLR4 by CHPV

**Figure 2:**
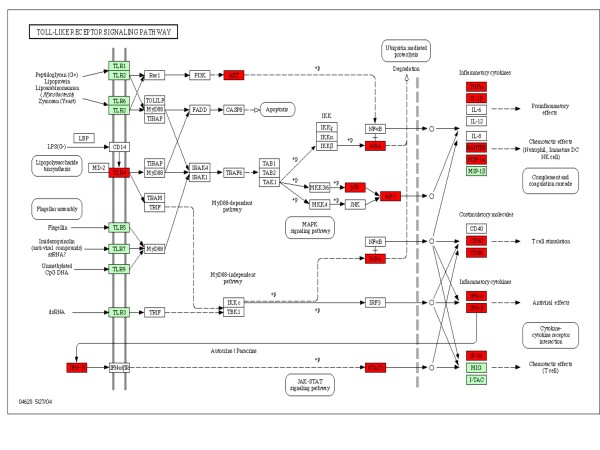
** Flow chart showing the genes involved in TLR signaling pathway.** In the TLRs signaling path way chart, genes highlighted in red boxes represent TLR4-related genes up-regulated in RAW cells during Chandipura viral infection. The other TLRs are highlighted in green boxes.

**Figure 3:**
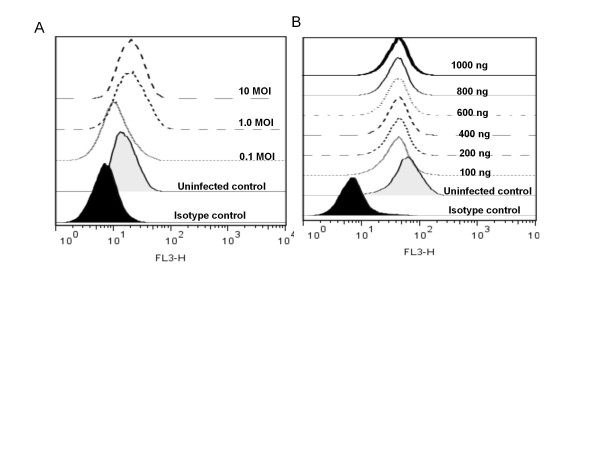
** Analysis of membrane expression of TLR4 in RAW cells.** RAW cells were infected with different MOI of Chandipura virus. In a second set of experiments, the cells were treated with different concentration of LPS. The cells were incubated for overnight at 37°C. The cells were then stained with an anti-mouse TLR4- PE-Cy7 conjugate and analyzed by flow cytometry. Half-offset histogram of mean fluorescent intensity of (A) RAW cells infected with Chandipura virus. (B) RAW cells treated with LPS.

**Figure 4:**
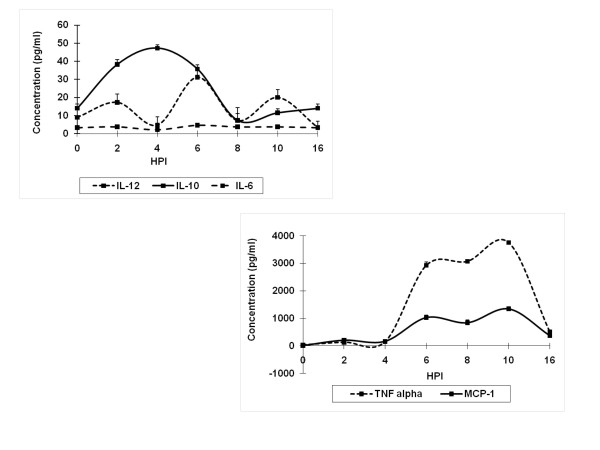
** Proinflammatory cytokine levels in culture supernatants of Chandipura virus-infected RAW cells.** RAW cells were infected with virus. The supernatant was collected on alternate hours up to 10 h PI. The final sample was collected 16 h PI. The levels of TNF-α, MCP-1, IL-10, IL-6, IL-12 and IFN-γ in the undiluted supernatant were quantified using a cytometric bead assay. The beads were acquired and analyzed using a FACSCalibur cell sorter and BD™ Cytometric Bead Array Software (BD Bioscience). The level of cytokines was quantified using known standard supplied with kit. Values represent the mean + standard error (SE) of triplicate experiments. IFN-γ could not be detected in the samples.

Gene regulation in RAW 264.7 cells during infection was studied using microarray techniques. The results indicated that several genes were up-regulated, and that the up-regulated genes were involved in TLR pathways, cytokine-cytokine receptor interactions, and the MAPK pathway (Table 1). Interestingly, up-regulated TLR genes were mainly involved in the TLR4 pathway (Figure 2).The up-regulation of TLR4 during infection was further validated in experiments studying regulation of membrane expression of TLR4 and secretion of proinflammatory cytokines in infected RAW cells. In this experiment LPS, a known activator of TLR4, was used as the positive control. RAW cells up-regulated TLR4 expression after infection with virus at 10 and 1.0 MOI, but down-regulated with 0.1 MOI (Figure 3A). Upon LPS treatment, the cells down-regulated TLR4 expression at all concentrations used in the experiment (Figure 3B). In the second experiment, the downstream signaling products of TLR4 (proinflammatory cytokines) were measured. Signature proinflammatory cytokines such as TNF-α, MCP-1, IL-12, IL-10, IFN-γ and IL-6 were measured in the supernatants of virus infected cells. Uninfected cell culture supernatants were used as control. Detectable levels of TNF-α and MCP-1 were observed 2 h PI (130 and 206 pg/mL, respectively) and reached a maximum at 10 h PI (3759 and 1348 pg/mL, respectively). IL-10 levels peaked at 4 h PI, and then gradually decreased to < 10 pg/mL by 8 h PI. Biphasic behavior was noticed with respect to IL-12 secretion. The level peaked at 2, 6, and 10 h PI and reached baseline at 4 and 8 h PI (Figure 4). No differences in IL-6 levels were observed. Similarly, no IFN-γ could be detected.

**Table 1:** Genes involved in different signaling pathways within Chandipura virus infected RAW cells

**Path ways involving up-regulated genes**	***P*-value**
Toll-like receptor signaling pathway	6.02E-12
Cytokine-cytokine receptor interaction	2.65E-08
MAPK signaling pathway	6.78E-06
Antigen processing and presentation	0.00175
Jak-STAT signaling pathway	0.00189
T cell receptor signaling pathway	0.00859
**Path ways involving down-regulated genes**	
Selenoamino acid metabolism	0.0257
Galactose metabolism	0.0291
Chondroitin sulfate biosynthesis	0.0345
Insulin signaling pathway	0.0375

### TLR4 in disease pathogenesis

**Figure 5:**
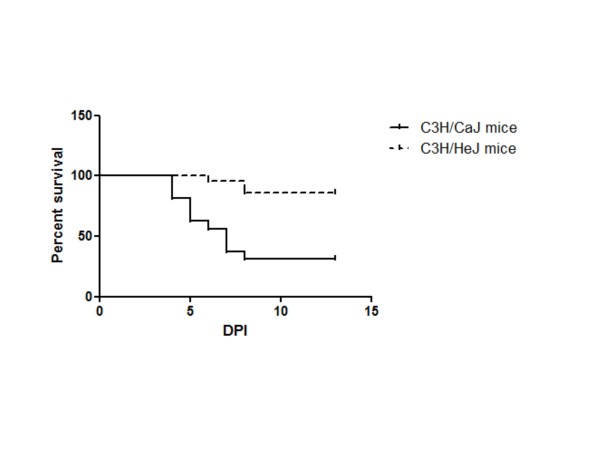
** Survival of virus-infected C3H/HeJ and C3H/CaJ mice.** Mice were infected with Chandipura virus. The infected mice were observed up to 13 days PI. The percent survival was calculated using Kaplan-Meier statistics and GraphPad Prism 5 software. Significantly improved survival rates were observed in C3H/HeJ mice (*P* < 0.000).

**Figure 6:**
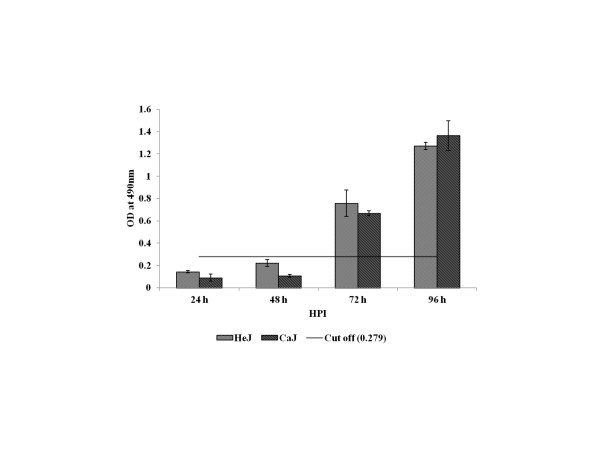
** Chandipura specific IgM in sera of infected C3H/HeJ and C3H/CaJ mice.** The level of IgM in the sera was determined by mouse IgM capture ELISA. The values are optical density (OD) at 490 nm wave length. The serum from three mice of infected as well as uninfected mice from C3H/HeJ and C3H/CaJ mice was processed separately in each time points. The values are Mean ± SE of three mice. Cut off value derived from mean OD of age matched uninfected control mice plus 3 standard deviation (SD).

**Figure 7:**
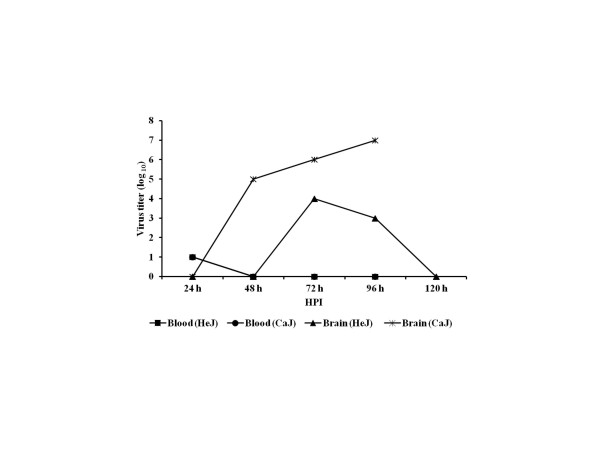
** Virus titer in blood and brain from infected C3H/HeJ and C3H/CaJ mice.** The sera and brain suspensions from three mice of C3H/HeJ as well as C3H/CaJ mice were titrated in vero E6 cells. Virus concentration was determined by end point dilution assay and reciprocal of highest dilution which showing 50% cell lysis was considered end point. The TCID_50_ per 100 μL was calculated by Reed and Munch formula. Each point in line graph represents mean virus titer of three mice.

**Figure 8:**
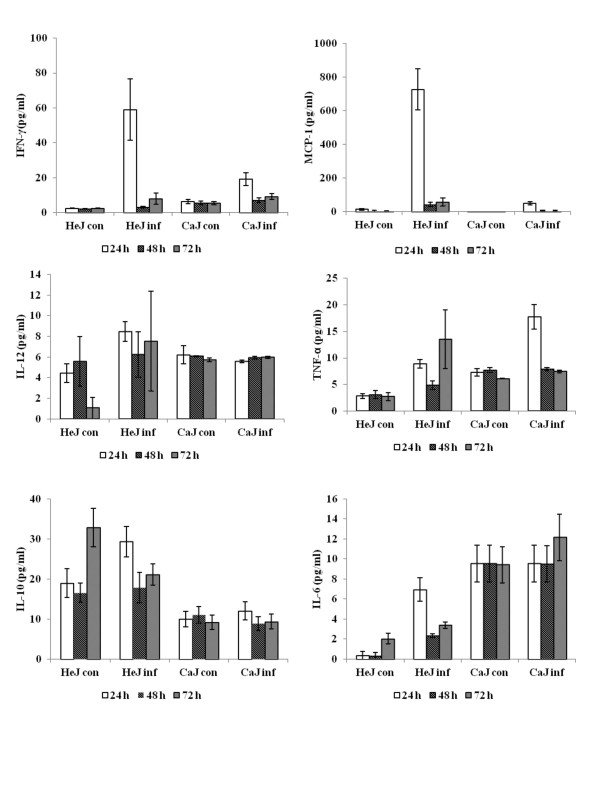
** Level of proinflammatory cytokines (TNF-**α**, IFN-**γ**, MCP-1, IL-6, IL-10 and IL-12p70) in sera of C3H/HeJ and C3H/CaJ mice**. At various time points, three mice from both infected (inf) as well as control (con) mice were bled. The sera were separated and used for cytokine quantification. The levels of TNF-α, MCP-1, IL-10, IL-6, IL-12 and IFN-γ in the undiluted sera were quantified using a BD™ Cytometric Bead Assay mouse inflammation kit. The beads were acquired and analyzed using a FACSCalibur cell sorter and BD™ Cytometric Bead Array Software (BD Bioscience). The level of cytokines was quantified using known standard supplied with kit. Values represent the mean ± SE of three mice.

To explore the possibility that TLR4 is involved in disease pathogenesis, wild-type C3H/CaJ mice and TLR4 mutant C3H/HeJ mice were infected with CHPV. Mean survival time and survival rate was calculated. Proinflammatory cytokines, virus specific IgM and virus titer was measured in these mice. The mean survival time was higher in C3H/HeJ mice (208.5 ± 4.6 h) than those in C3H/CaJ mice (160.5 ± 11.56 h). Similarly, the mean survival rate of C3H/HeJ mice was significantly higher than that of wild-type C3H/CaJ mice (86% vs 31.25%, *P* < 0.000; Figure 5). Gradual increase in virus specific IgM level was observed in both mutant and wild-type mice. However, no significant difference in IgM level was noticed between these groups (Figure 6). The virus titer in brain was gradually increased from 10^4^ at 48 h PI to 10^7^ TCID_50_/mL at 96 h PI in C3H/CaJ mice. Titration was not done beyond 96 h PI as mortality started at this time point. In contrast, in C3H/HeJ mice, the virus titer was 10^4^ TCID_50_/mL at 72 h PI and reached undetectable level at 120 h PI (Figure 7). In blood, the virus titer was 10^1^ TCID_50_/mL at 24 h PI and it was undetectable level in all other PI hours tested. The level of MCP-1 and IFN-γ cytokines was significantly higher in infected C3H/HeJ mice than those in infected C3H/CaJ mice. The level of MCP-1 in C3H/HeJ mice (726 ± 122 pg/mL) at 24 h PI was greater than that in C3H/CaJ mice (50 ± 9.6 pg/mL). Similarly, IFN-γ level was higher in C3H/HeJ mice (59 ± 17 pg/mL) than that in C3H/CaJ mice (19 ± 3.9 pg/mL) (Figure 8). Significant level of difference in quantity was not observed in other cytokines (IL-12, IL-6, IL-10, TNF-α) tested.

### Nitric oxide in disease pathogenesis

**Figure 9:**
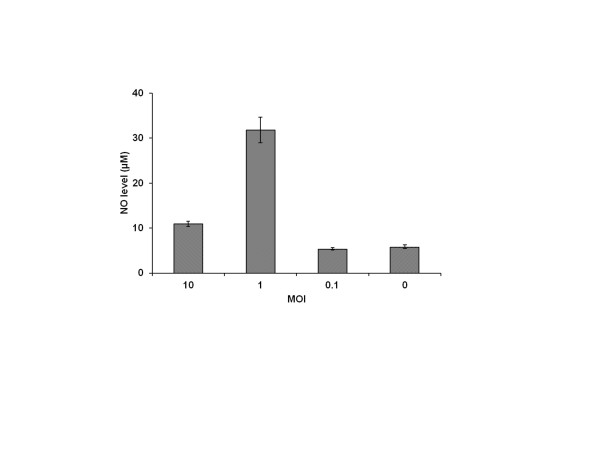
** NO levels in culture supernatants of Chandipura virus-infected RAW cells**. RAW cells were infected with different MOI of virus. The supernatants were collected at 24 h PI. The NO level in the supernatants was measured using the Griess method. Values represent the mean ± SE of triplicate experiments.

**Figure 10:**
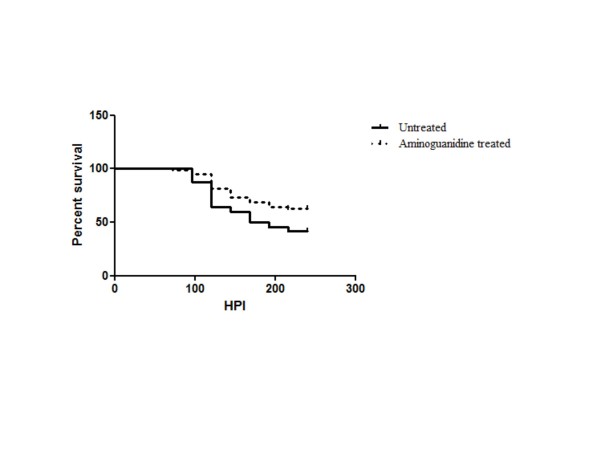
** Survival of virus-infected mice treated with the NO inhibitor, AG**. Young Swiss albino mice were infected with Chandipura virus. One group of infected mice was treated with AG. Non-AG-treated mice were used as a virus control. The treatment was continued up to 120 h PI. Mortality was observed up to 240 h PI. Percent survival was calculated using Kaplan-Meier statistics and GraphPad Prism 5 software.

CHPV infected RAW cells secreted NO and NO level was higher at 1.0 MOI (31 μM) than at 10 MOI (10 μM) at 24 h PI (Figure 9). The level was similar in 0.1 MOI and uninfected cell culture supernatants. To ascertain the role of NO in disease pathogenesis, virus infected Swiss albino mice were treated with the NO inhibitor, AG. Partial protection was observed in AG-treated mice (62%) as compared to that in AG untreated mice (42%). The mean survival time for AG treated group (203 ± 7 h) was significantly higher than control group (180 ± 7 h; *P* < 0.05; Figure 10).

## Discussion

In the current study, the role of TLRs in the response to CHPV in RAW cells was examined. RAW 264.7 cells express various TLRs and have a high sensitivity to TLR ligands [29]. We demonstrate that CHPV infection regulates the TLR4 during infection in RAW cells and TLR4 and NO involved in disease pathogenesis in mice.

TLR4 is involved in LPS signaling and senses Gram negative bacteria [30,31]. The role of TLR4 in LPS signaling has been reviewed in detail elsewhere [32,33]. The first indication that TLR4 was involved in the response to viral infection came from research on RSV [34] and VSV [35]. We compared our results with available literature on vesicular stomatitis virus and other viruses. In experimental HSV-1 infection in TLR2 knockout mice, it was reported that TLR2 mediated cytokine response in the brain contributes to the death of the mice [36]. In our study, we used TLR4 mutant, C3H/HeJ mice to demonstrate the role of TLR4 in disease pathogenesis. C3H/HeJ mice are highly resistant to LPS, showing no usual biological effects, yet, they show a normal response to other bacterial products and to most cytokines induced by LPS. The LPS-resistant phenotype results from defects in the gene for TLR4 (*Tlr4*) [30,31]. The percentage survival rate and mean survival time was significantly higher in TLR4 mutant C3H/HeJ mice as compared to those in wild type C3H/CaJ mice. Initially, we suspect that a proinflammatory cytokines level is associated with C3H/HeJ survival. In order to confirm that six signature proinflammatory cytokines in infected C3H/HeJ and C3H/CaJ mice were quantified. Interestingly, the level of MCP-1 and IFN-γ was significantly higher in CHPV infected C3H/HeJ mice than those in infected C3H/CaJ mice.

The role of MCP-1 in protection against virus infection was well documented in influenza virus infection in mice model [37]. The authors opined that lack of MCP-1 responsible for increased virus load in virus infected mice. In our different study, it was observed that high level of MCP-1 in patients recovered from CHPV infection (data not shown). Similarly IFN-γ is reported to be a critical antiviral mediator to eliminate the virus from CNS [38]. IFN-γ treatment at early stage of influenza virus infection protects mice from death [39]. IFN-γ effectively inhibits the replication of VSV in neuronal cells [40]. These cytokines might contribute increase in survival rate in C3H/HeJ mice. These findings could be verified by experimental animal model using MCP-1 and IFN-γ deficient mice. In the current study, there was no significant difference in the level of virus specific antibodies between C3H/HeJ and C3H/CaJ mice which eliminate the possibility of difference in level of antibodies in protection against CHPV infection.

It was reported that IFN-γ treatment enhances the secretion of NO in VSV infected neuronal cells which inhibit the virus replication [41]. In this study, we demonstrate that CHPV infection in RAW cells induced NO secretion. As the NO inhibits the virus replication, it was thought that NO inhibitor (AG) treatment may increase the severity of pathogenesis in infected mice. Conversely, partial protection was observed in treated mice. Thus, we concluded that NO may contribute to the pathogenesis and mortality in infected mice.

## Conclusions

Chandipura virus activates TLR4, which leads to the secretion of proinflammatory cytokines and NO. Despite activation of the innate immune system, mortality observed in young mice. Partial protection in TLR4 mutant mice and nitric oxide inhibitor treated wild-type mice indicated that TLR4 and NO contributed in disease pathogenesis. These results are significant in clinical management and designing antiviral intervention.

## Competing interests

The author(s) declare that they have no competing interests.

## Authors’ contributions

BA designed the study, performed the experiments and drafted the manuscript. PS helped with the experiments. All the authors read and approved the final manuscript.

## Pre-publication history

The pre-publication history for this paper can be accessed here:

http://www.biomedcentral.com/1471-2334/12/125/prepub
